# Long-Term Survival of *Mycobacterium avium* subsp. *paratuberculosis* in Fecal Samples Obtained from Naturally Infected Cows and Stored at −18°C and −70°C

**DOI:** 10.4061/2011/341691

**Published:** 2011-08-11

**Authors:** Eran A. Raizman, L. A. Espejo, S. J. Wells

**Affiliations:** ^1^320 Fernleaf Dr West Lafayette, IN 47906, USA; ^2^Department of Veterinary Population Medicine, College of Veterinary Medicine, University of Minnesota, St. Paul, MN 55108, USA

## Abstract

The objective was to evaluate the survival of Mycobacterium paratuberculosis (Map) in naturally infected dairy cows feces under long periods of freezing at −18°C and −70°C. Samples were collected from cows previously tested positive with serum ELISA or fecal culture, or with clinical signs of Johne's disease. Samples were stored at −18°C and/or −70°C and recultured in Herrold's egg yolk media every 3–6 months. A proportional odds mixed model was used for data analysis. Sixty nine fecal samples were stored for different periods between September 2002 and January 2005. Of these, 45 (65%) were stored at −18°C and 24 (35%) at −70°C. Average number of days between repeated culture dates was 98 and 84 for −18°C and −70°C, respectively. Median number of repeated cultures was 6 and 4 for samples stored at −18°C and −70°C, respectively. After adjusting for initial sample bacterial load, the effects of temperature or number of thawing and refreezing cycles on Map viability were not significant. The probability that a sample decreases from high to moderate-low bacterial load and from moderate-low to negative bacterial load was 13.5% per month. Although this study found gradual reduction of Map concentration in stored fecal samples through time, overall survival in −18°C can ease fecal samples management in laboratories with low-processing capacity or lack of −70°C freezer.

## 1. Introduction

*Mycobacterium paratuberculosis* (Map), the causative agent of Johne's disease (JD) in domestic and wild ruminants, is known to survive for long periods of time in different environmental conditions [1, 2]. This is especially true in the dairy farm environment near shaded animal management locations where frequent introductions of manure are occurring [3, 4]. Several reports describe long-term *in vitro *Map survival under various physical and environmental conditions including survival in water, urine, and manure and at extreme temperatures [3, 5, 6]. The organism was found to survive in pond water for up to 270 days under *in vitro* conditions and up to 246 days in bovine feces under natural conditions where environment temperature varied between −3°C and 23°C [7]. Viable bacteria were isolated after storing the same samples for five months at −14°C, then at 4°C for five months and finally at 38°C for eight months. Bacteria stored for 12 months at −14°C and then at 4°C for five months were also viable [5].

The value of fecal culture in the diagnosis of JD in livestock and especially in cattle has long been established [8]. Fecal samples are normally collected directly from the rectum or from the environment and transported to the laboratory for further processing. Limited processing laboratory capacity may hinder the ability to test all samples immediately. One solution that has been proposed to overcome this problem is to store the specimens at −70°C until facilities are available to complete the sample processing [9]. This would allow a single collection of samples, regardless of herd size or when the laboratory is ready to process the samples, assuming that storage space is available. Furthermore, sample storage can also be used for research studies performed over a long period of time. However, limited information is available on the viability of Map under these conditions and most studies have used relatively small sample sizes of fecal specimens [9–11]. In a recent study using 11 cows, it was found that the samples storage at −20°C for one week had adverse effects on the viability of Map, whereas short-term storage of fecal samples at 4°C (48 hours to one week) and longer-term storage (3 months) at −70°C had no negative effect on Map survival [10]. Thus, while the authors evaluated Map viability under different shipping and storage conditions, storage was evaluated only up to 3 months. Other than this study, the impact on Map survival of long-term storage under −18°C has not been evaluated, as far as the authors could assess in the published literature. Long-term viability of Map under these conditions can often reduce laboratory costs significantly for JD research. This is especially true in laboratories where the availability of −70°C freezers is very limited due to their high cost. Therefore, the objective of this study was to evaluate the survival of Map in manure from naturally infected cows over long periods of freezing at −18°C and −70°C. Our hypothesis was that Map long-term viability at −18°C and −70°C would not be significantly different.

## 2. Material and Methods

We used a targeted sampling approach where fecal samples were collected from cows known to be infected with JD through previous serum ELISA or fecal culture testing. We also sampled cows with clinical signs consistent with JD (i.e., chronic diarrhea, progressive weight loss, and reduced milk production) regardless of previous fecal culture results. Fecal samples were collected using a disposable obstetric glove and placed in a 40 mL plastic container for transportation to the laboratory in an iced cooler. A portion of each sample was sent for initial fecal culture, and the rest was divided into two equal portions and stored at −18°C and −70°C in a plastic container appropriately labeled with the sample ID, herd name, date of collection, and date of last culture. When manure sample was too small to be divided, priority was made to store samples at −18°C over −70°C. Our sample size included a total of 51 cows from 17 herds. Of these, 35 cows were sampled once, 27 (77%) at −18°C and 8 (23%) at −70°C. Fourteen cows were sampled twice; one of the pair samples was stored at −18°C and the other 13 at −70°C. Two cows were sampled 3; times of these, one was stored only at −18°C and the other two at both −18°C and −70°C. Samples were recultured approximately every 3–8 months. All samples were processed at the Minnesota Veterinary Diagnostic Laboratory using a previously described bacterial culture method [12]. Briefly, a sedimentation culture procedure was used with 72 hours of sedimentation prior to inoculation of 4 tubes containing Herrold's egg yolk medium. Colony counts were recorded weekly for 16 weeks, and final results were reported as negative, light (mean of 0.25 to 9 colonies/tube (CPT)), moderate (mean of 10 to 49 CPT), moderate-heavy (mean of 50–100 CPT), and heavy (>100 CPT) bacterial load. Each sample was recultured until results were negative in two consecutive cultures or until the fecal material was exhausted.

## 3. Statistical Analysis

All statistical analysis was performed using SAS (SAS Institute version 9.1). 

To test our hypothesis, a proportional odds mixed model approach (PROC GLIMMIX) was used. We assumed a multinomial distribution for the response variable with 3 levels (0 = negative fecal culture, 1 = moderate-low fecal shedding (1–50 CPT), and 2 = high fecal shedding (>50 CPT)). Independent variables were time in months (continuous) which is the total time that samples were kept in the freezer, storage temperature (dichotomous) (−18°C or −70°C), and initial culture result (ordinal categorical with 4 levels of fecal bacterial load). Each fecal sample had a different time to testing; therefore, the model assumed repeated measures (fecal culture) for the same experimental units (fecal samples) and testing time was not constant among fecal samples. As a result, time of testing was also included in the model as a categorical random variable. Because some of the cows have more than one sample tested, we used a nested structure of the data, which is sample nested within cow. In order to test the effect of the number of thawing and refreezing cycles of the samples on Map viability, we ran the same model described above once together with the main effect (months) and then as a main effect. In the final model, a *P* value of <0.05 was considered statistically significant. 

## 4. Results

A total of 69 samples from 51 cows were stored for different periods between September 2002 and January 2005. Of these, 45 (65%) were stored at −18°C and 24 (35%) at −70°C. Map was cultured at the initiation of the study from all 69 samples. Of the 45 samples stored at −18°C, 35 (78%) and 10 (22%) samples started the study with high and moderate-low bacterial load, respectively. Of the 24 samples stored at −70°C, 21 (88%) and 3 (12%) were high and moderate-low bacterial load, respectively. The average number of days between repeated cultures was 98 (minimum = 40, maximum = 128) and 84 (minimum = 67, maximum = 207) for the −18°C and −70°C storage temperatures, respectively. The median number of repeated cultures was 6 (maximum = 13, minimum = 2) and 4 (maximum = 9, minimum = 2) for samples stored at −18°C and −70°C, respectively. Before adjusting for initial sample bacterial load, the median survival days was 549 (maximum 1016, minimum = 124) and 740 (maximum = 1042, minimum = 112) for samples stored at −18°C and −70°C, respectively. This difference was statistically significant (*P *< 0.01; Table 1). After adjusting for initial fecal sample bacterial load, the temperature effect was not statistically significant (*P *= 0.084). Similarly, the effect of the number of thawing and refreezing cycles on Map viability was not found to be statistically significant (*P =* 0.71) and hence was not included in the final model. The overall estimated Odds Ratio for bacterial load reduction for each month in the freezer was 1.13 (95% CI 1.01 and 1.20) per month in the freezer; therefore, the probability that a sample viability decreases from moderate-low to bacterial load or from low to negative bacterial load was 13% per month. Because the proportional odds model did not converged when the interaction temperature of storage by time was included, the analysis was stratified by storage temperature to evaluate the effect of time in months on the probability of decreasing one category the bacterial culture results. A similar proportional odds model used for the full data set was fitted separately for samples storage at −18 and −70. The results of the model indicated that the probability of decreasing one category the bacterial culture result was 13% (95% CI 8.3–19.4; OR = 1.13 95% CI 1.08–1.19) for samples storage at −18 and 14.7% (95% CI −22.4–70.4; OR = 1.15 95% CI 0.78–1.70), for samples storage at −70, after adjusting by initial bacterial culture result in both models. 

## 5. Discussion

The current study represents the first evaluation of the long-term viability of Map in fecal samples from naturally infected cows when stored at −18°C and −70°C, using a relatively large sample size and more than two years of storage time. Previous studies used a small sample size over a shorter time period [9–11]. In our study, Map survived at −18°C on average 540 days (i.e., 18 months). This period of time is sufficient to allow low capacity laboratories as well as laboratories without −70°C freezers the ability to perform Map testing and research. Our findings suggest that overall Map viability on the long term was not significantly affected by the number of thawing and refreezing cycles compared to storage time. It is possible, however, that, with a larger sample size, a significant impact would be assessed. The methodology used in this study, however, was not designed to address the specific issue of thawing and refreezing. The authors acknowledge that to most accurately assess freeze thaw, aliquots should be made at the beginning of the study where some aliquots would be subjected to freezing and thawing cycles while others are continuously frozen until the date of culture. 

Giving the long-term survival in both temperatures, this can ease sample management for long-term research using either −18°C or −70°C. It is important to consider, however, that our sampling targeted high bacterial load cow shedders and results may not apply to low-to-moderate shedders, which are more commonly found during routine herd testing. Additional research is required on a larger sample size of low-to-moderate bacterial load fecal samples to determine whether long-term storage is a viable option for these samples as well. While the freezing and thawing cycles in our study do not exactly mimic these cycles in the nature, our study results provide additional evidence of the ability of Map to survive extreme environmental conditions on farms. It is important to consider that daily cycles of thawing and refreezing are common in the northern latitudes of the north hemisphere. 

While a clear tendency for decreasing Map viability over time was observed in the current study, several samples resulted in an increased bacterial load between two consecutive cultures. We do not have a definitive explanation for this lack of repeatability, but it is plausible that fecal sample homogeneity was not the same in each sample processing, perhaps due to non uniform distribution of Map within the sample. This is, however, in contrast to what was reported in previous study which showed that the distribution of Map cultivable quantities was relatively uniform in fecal samples obtained from both subclinical and clinical animals [13]. The authors, however, did not quantify fecal bacterial load as we did in the current study.

A proportional odds model (also known as ordinal logistic regression) was chosen to analyze the data because the results of the fecal culture are normally classified in categories (negative, low, and high) rather than the average of colonies per tube. This classification system provides information about not only the shedding status of the cows, but also the risk of the disease transmission. An important assumption of this type of model is the proportionality of the odds for each level of the outcome fecal culture result. That assumption was initially evaluated using the Chi-square score test and plotting the odds against the fixed effects. Data could also be analyzed using a nominal logistic regression, where there is no assumption of proportionality of the odds; however, that type of model requires even larger amount of data because more parameters need to be estimated. In our model, we tested the effect of the initial culture result on the probability of a subsequent decrease in bacteria cultured (response 0, 1, and 2). Samples with high bacterial load on the first fecal culture after collection had a higher probability of reduction in Map concentration than samples with moderate-low initial bacterial load. In other words, the proportion of bacterial death that must occur in order to decrease the fecal culture bacterial load from moderate-low to negative is smaller than the proportion of bacterial death that must occur in order to decrease fecal culture bacterial load from high to moderate-low. Therefore, the odds of decreasing the culture are higher on moderate-low initial bacterial load cultures. While a recent study addressed different temperatures and shipping methods, different initial bacterial loads were not addressed at all [10]. Additionally, our stratified analysis indicates that the odds for reduction in Map viability overtime was statistically significant for samples stored in −18°C but not significant for samples stored at −70°C. This is an important fact to consider when the use of either freezer is considered whether for research or diagnostics. 

The current study was a natural experiment, and the weaknesses of the design were addressed on the analysis when possible. Nevertheless, the main weakness of this study is the lack of constant time interval between repeated cultures and that a different number of samples was used in the two storage temperatures. We believe, however, that the lack of constant time interval was addressed in the statistical model by using a random intercept for storage time and hence did not affect the results of this study. Also, a targeted sampling approach was used in this study and samples were not randomly selected from cows. However, we do not believe that this affected the study results since the subject of the study was the Map organism independent of the animal it was shed from. Additionally, to overcome possible biased estimates due to nonrandom allocation of the samples between the two treatments, we included the variable of the initial culture results to control/adjust for nonrandom allocation of the samples based on initial fecal culture results. Despite that in our analysis we controlled for possible known biases by adjusting for those variables that could not be controlled on the study design, it was impossible to control for unknown sources of biases such as random allocation does.

In summary, this study has shown that Map can survive over long periods (median approximately 18 months) at both −18°C and −70°C and the difference in survivorship between both storage temperatures was shown not to be statistically significant. Nevertheless, Map in samples storage at −70°C showed a trend for twice (OR = 2.2) longer survival which can result from the relatively small sample size stored at −70°C. This implies that fecal samples can be stored for long periods in case a laboratory does not have the capacity to process all the samples at one time or if a laboratory does not have a −70°C freezer. Additionally, the results of this study are an additional indication that Map has the ability to survive long periods of time under extreme environmental conditions that can be found on many dairy farms and crop fields. Our study has shown that this ability, however, depends on the initial bacterial load of the manure. Future studies need to address Map survival with low initial bacterial load (0.25–10 CPT) and to perform the analysis using the real number (continuous) CPT rather than the categorical culture result, which will determine the survival curve against storage time per treatment.

## Figures and Tables

**Table 1:** Parameter estimates (standard error—SE, odds ratio—OR and the OR 95% confidence interval—CI) of the proportional odds mixed model for changes on the fecal culture results across time for *Mycobacterium avium *subsp*. paratuberculosis* in dairy cows in Minnesota, USA.

Parameter	Levels	Estimate	SE	OR	CI	*P* value
Intercept*	0	−6.44	0.54			<0.01
	1	−2.43	0.34			<0.01
	2	Reference	—	—	—	—
Months, n	1	0.13	0.02	1.14	1.10, 1.18	<0.01
Temperature, C°	−18°	0.79	0.31	2.20	0.82, 5.99	0.08
	−70°	Reference	—	—	—	—
Initial bacterial load**	1	4.38	0.56	79.80	13.4, 478.02	<0.01
	2	2.47	0.53	11.82	2.21, 62.97	0.02
	3	1.65	0.46	5.18	1.19, 22.85	0.04
	4	Reference	—	—	—	—

*0: negative, 1: mean of 0.25–50 colonies/tube (CPT)), and 2: mean of >50 CPT.

**1: mean of 0.25–9 colonies/tube (CPT)), 2: mean of 10–49 CPT, 3: heavy (mean of 50–100 CPT), and 4: >100 CPT.
